# The role of BRD7 in embryo development and glucose metabolism

**DOI:** 10.1111/jcmm.12907

**Published:** 2016-07-22

**Authors:** Yoo Kim, Mario Andrés Salazar Hernández, Hilde Herrema, Tuncay Delibasi, Sang Won Park

**Affiliations:** ^1^ Division of EndocrinologyBoston Children's HospitalHarvard Medical SchoolBostonMAUSA; ^2^ Department of Internal MedicineSchool of Medicine, KastamonuHacettepe UniversityAnkaraTurkey

**Keywords:** bromodomain‐containing protein 7, embryogenesis, glucose metabolism

## Abstract

Bromodomain‐containing protein 7 (BRD7) is a member of bromodomain‐containing protein family and its function has been implicated in several diseases. We have previously shown that BRD7 plays a role in metabolic processes. However, the effect of BRD7 deficiency in glucose metabolism and its role in *in vivo* have not been fully revealed. Here, we report the essential role of BRD7 during embryo development. Mice homozygous for BRD7 led to embryonic lethality at mid‐gestation. Homozygous BRD7 knockout (KO) mice showed retardation in development, and eventually all BRD7 KO embryos died *in utero* prior to E16.5. Partial knockdown of *Brd7* gene displayed mild changes in glucose metabolism.

## Introduction

Bromodomain‐containing proteins (BRDs) are evolutionarily highly conserved protein modules, which contain a hydrophobic binding site for acetylated lysine residues, which that are often present on chromatin‐associated proteins and transcriptional factors, such as histone acetyltransferases and methyltransfereases 1, 2. Previous studies have shown that defects in proteins with BRDs lead to the development of various diseases. For instance, chromosome mapping has revealed that an interruption in the coding sequence of bromodomain on chromosome 15 and 19 is responsible for the development of midline carcinoma, a lethal squamous cell cancer 3, 4. The involvement of BRDs in the disease biology was also implicated in acute myeloid leukaemia and B cell lymphoma 4, 5, 6. In addition, it was shown that polymorphism in the bromodomain motif of the switch/sucrose non‐fermentable (SWI/SNF) related, matrix associated, actin dependent regulator of chromatin, subfamily A, member 2 (SMARCA2), which encodes Brahma in the SWI/SNF chromatin‐remodelling complex, is associated with the development of schizophrenia 7. Furthermore, cAMP response elements binding protein‐binding protein (CBP) is a bromodomain containing enzyme that is involved in histone acetylation. It was shown that the alteration in CBP activity is involved in neurodegenerative processes, which eventually leads to neuronal disorders, such as Huntington's disease, Alzheimer's disease, or Rubinstein‐Taybi syndrome 8, 9.

Bromodomain‐containing protein 7 (BRD7) is a member of the bromodomain‐containing protein family and it is ubiquitously expressed in many tissues, including liver, brain, heart, lung, colon, and skin, as well as tissues and cells in reproductive, urinary tract, and immune systems. BRD7 was shown to be involved in inhibition of cell cycle and cellular growth by mitogen‐activated protein kinase, extracellular signal‐regulated kinase, and retinoblastoma/E2F pathways 10, 11, 12, 13, 14, 15. Accumulating studies have shown that BRD7 is involved in human cancers. The expression of BRD7 was found decreased in nasopharyngeal carcinoma 12. More recently, BRD7 has been suggested to play a role as a potent *tumor* suppressor by binding to p53 and breast cancer gene 1 (BRCA1) 13, 14, 15.

Meanwhile, our recent study has shown that BRD7 is involved in the regulation of glucose metabolism and insulin signalling pathway 16. We have shown that BRD7 interacts with the regulatory subunits of phosphatidylinositol 3‐kinase (PI3K), p85α and p85β, and increases the nuclear translocation of p85s 16, 17. We have previously demonstrated that p85s interact with the spliced form of X‐box binding protein 1 (XBP1s), a master regulator of endoplasmic reticulum (ER) folding activity, and increase the nuclear translocation of XBP1s 18. Accordingly, we have shown that BRD7 leads to increased the nuclear translocation and transcriptional activity of XBP1s 16. Of note, we have discovered that the hepatic expression levels of BRD7 are significantly reduced in both genetically obese and high fat diet (HFD) induced obese mice 16. We have shown that reduced BRD7 levels in obesity are an important underlying mechanism for the development of ER stress, glucose intolerance, and type 2 diabetes in obesity 16. We have documented that acute up‐regulation of BRD7 in the liver of obese mice reinstates XBP1s nuclear translocation and re‐establishes glucose homoeostasis in obesity 16.

Embryonic lethality is a common phenomenon that often occurs in genetically engineered mice. Lethality during gestation provides information on the importance of the target gene during embryogenesis and whether it is an essential genetic factor that affects other components. In this report, we used heterozygous BRD7 whole body knockout (KO) mouse model to investigate the role of BRD7 in embryonic development. Here, we report homozygous BRD7 whole body KO mice are embryonic lethal during gestation development. We further investigated the effects of reduced hepatic BRD7 expression levels on the whole body glucose metabolism.

## Materials and methods

### BRD7 heterozygous KO mice

The heterozygous BRD7 whole body KO mouse line (BRD7^+/−^), 129‐BRD7<tm2a(EUCOMM)Wtsi>/WisiH, was purchased from the European Conditional Mouse Mutagenesis Program (EUCOMM). The mice were generated by the insertion of two reporter genes of β‐galactosiase (LacZ) and neomycin‐resistance gene cassette, flanked by FRT sites, which creates a frame shift and disrupts gene function. Introduction of Flp Recombinase can converts the knockout allele to a conditional allele and restores the expression of *Brd7* gene. Cre Recombinase deletes the critical region within *Brd7* exons to generate a frameshift mutant. All BRD7^+/−^ mice in this study had a mixed background with the 129 Sv/J and C57BL/6J strains and they were bred with C57BL/6J for at least 15 generations. We determined genotypes by performing PCR of genomic DNA isolated from tail biopsies. The PCR conditions were as follows: 94°C for 10 min., 30 cycles of 94°C for 30 sec., 58°C for 30 sec., and 72°C for 30 sec.; and 72°C for 5 min. We used the following primers for genotyping. Bromodomain‐containing protein 7 wild‐type Forward 5′‐GTGACTTACATCCCCGGAGC‐3′, BRD7 wild‐type Reverse 5′‐TTAGAGGAGTAGCCTTCCGTGAG‐3′, INS Forward 5′‐AGGCGCATAACGATACCACGAT‐3′, INS Reverse 5′‐CCACAACGGGTTCTTCTGTT‐3′.

### Embryo analysis

For embryonic study, we interbred the heterozygous BRD7 whole body KO mice. Pregnant females were euthanized by CO_2_ and embryos were dissected in PBS under the confocal microscope (Leica, Mannheim, Germany). Genomic DNA was extracted in 50 μl of lysis buffer (25 mM NaOH and 0.2 mM ethylenediaminetetraacetic acid) by boiling at 100°C for 20 min. After cooling down to room temperature, 50 μl of 40 mM Tris‐HCL was added, followed by centrifugation at 16,000 × g for 1 min. Genotyping of embryos was done by performing PCR as described above in BRD7 heterozygous KO mice section, using the same primer sets, BRD7 WT and INS.

### Cell culture

Mouse embryonic fibroblast (MEF), HEK293, and 293A cells were cultured in DMEM with 10% fetal bovine serum (FBS), 10 U/ml penicillin and 1 μg/ml streptomycin. Cells were maintained at 37°C in a 5% CO_2_ humidified atmosphere.

### Production and transfection of siRNA

*Brd7* specific siRNA was purchased from Ambion (Life Technologies, Beverly, MA, USA). The sequences of siRNA are as follows: Sense 5′‐GAGUCAAGGAGGAUAAAAAtt‐3′ and Antisense 5′‐UUUUUAUCCUCCUUGACUCtt‐3′. Bromodomain‐containing protein 7 siRNA was transfected into MEFs and HEK293 cells using Lipofectamine RNAiMAX Reagent from Invitrogen (Life Technologies) according to manufacturer's instruction.

### Production of shRNA adenovirus

The shRNA sequences for *Brd7* in pAD vector were as follows: BRD7shRNA Forward 5′‐CACCGGGCCTGGCTACTCCATGATTATCGAAATAATCATGGAGTAGCCAGGC‐3′ and Reverse 5′‐AAAAGCCTGGCTACTCCATGATTATTTCGATAATCATGGAGTAGCCAGGCC‐3′. pAD‐BRD7shRNA and lipofectamine were each diluted in 250 μl of OptiMEM and incubated at room temperature for 5 min. The DNA and lipofectamine in OptiMEM were combined and incubated at room temperature for 30 min. The DNA‐lipofectamine was diluted in 500 μl of OptiMEM and total of 1 ml of solution was added to 293A cells with 1 ml of OptiMEM in 6‐well plate. After 2 hrs of incubation, 2 ml of DMEM with 20% FBS was added to the cells. After 16 hrs of incubation, the media was changed to fresh DMEM with 10% FBS. The following day, the cells were transferred to 10 cm^2^ cell culture treated plate, and media was changed every 2 days until adenovirus was produced.

### Injection of BRD7siRNA and Ad‐BRD7shRNA

For BRD7siRNA administration, 250 nmol of siRNA was resuspended in 1 ml of distilled water. 250 μl of siRNA was mixed with 250 μl of complexation buffer provided by Invivofectamine 2.0 Reagent (Ambion, Life Technologies). The siRNA mix was added to 500 μl of Invivofectamine. After 30 min. of incubation, the mix was brought up to the total volume of 14 ml with PBS, and it was added to prewashed Amicon Ultra‐15 centrifugal filter device, followed by centrifugation at 4000 × g for 1 hr. siRNA was concentrated to the total volume of 500 μl of siRNA and 100 μl was used to inject mice *via* the tail vein. For injection of adenovirus, the viruses were diluted to a final volume of 100 μl with saline. Mice were put in a restrainer and the tail was heated with a heating lamp for vasodilatation. Adenovirus was injected to mice through the tail vein with a 28‐gauge needle. Following injection, mild pressure was applied at the injected spot to prevent bleeding.

### Quantitative real‐time PCR

We extracted total RNA using Trizol reagent according to the manufacturer's instructions (Invitrogen, Carlsbad, CA). We then obtained cDNA from RNA by conducting reverse transcription with 1 μg of RNA using iScript^™^ cDNA synthesis kit from Bio‐Rad (Hercules, CA, USA). The conditions of reverse transcription were as follows: 25°C for 5 min., 42°C for 30 min. and 85°C for 5 min. Quantitation of the targeted gene expression levels was conducted by SYBR‐based real‐time PCR assay on QuantStudioTM 6 Flex Real‐Time PCR system (Life Technologies). *18s ribosome* or β*‐actin* RNA level was used to normalize the gene expressions. We used the following primers to perform qPCR. 18s Forward 5′‐AGTCCCTGCCCTTTGTACACA‐3′, 18s Reverse 5′‐CGATCCGAGGGCCTCACTA‐3′, β‐actin Forward 5′‐TACCACCATGTACCCAGGCA‐3′, β‐actin Reverse 5′‐CTCAGGAGGAGCAATGATCTTGAT‐3′, BRD7 Forward 5′‐ATGGGCAAGAAGCACAAGAA‐3′, BRD7 Reverse 5′‐CCATGGGAAGATGTTCTGGG‐3′, glucose‐6‐phosphatase (G6P) Forward 5′‐CCGGTGTTTGAACGTCATCT‐3′, G6P Reverse 5′‐CAATGCCTGACAAGACTCCA‐3′, glucokinase (GCK) Forward 5′‐GAAAAGATCATTGGCGGAAA‐3′, GCK Reverse 5′‐CCCAGAGTGCTCAGGATGTTAAG‐3′, fructose1,6‐bisphosphatase (FbP) Forward 5′‐CTTTTTATACCCCGCCAACA‐3′, FbP Reverse 5′‐TCTTCAGAGGACCCCATGAC‐3′, phosphoenolpyruvate carboxykinase (PEPCK) Forward 5′‐TGACATTGCCTGGATGAAGT‐3′, PEPCK Reverse 5′‐GTCTTAATGGCGTTCGGATT‐3′, peroxisome proliferator‐activated receptor γ coactivator‐1α (PGC1α) Forward 5′‐TGATGTGAATGACTTGGATACAGACA‐3′, PGC1α Reverse 5′‐CAATGCCTGACAAGACTCCA‐3′, *tumor* necrosis factor α (TNF‐α) Forward 5′‐CCCTCACACTCAGATCATCTTCT‐3′, TNF‐α Reverse 5′‐GCTACGACGTGGGCTACAG‐3′.

### Blood glucose level measurement and blood collection

The blood glucose level was measured from the tail by clipping and using a glucose metre Contour (Bayer Heathcare LLC, Mishawaka, IN, USA). The blood was collected from the tail vein in heparin‐treated capillary tubes and put on ice until centrifugation. The collected blood samples were centrifuged at 8000 × g for 20 min. at 4°C.

### Glucose tolerance test and insulin tolerance test

For glucose tolerance test (GTT), animals were fasted for 16 hrs (5:00 p.m.–9:00 a.m.), and then subjected to baseline measurement of blood glucose levels from the tail, which we noted as 0 min. time‐point. Subsequently, d‐glucose solution (1.0 g glucose/kg bw for HFD fed mice and 2.0 g glucose/kg bw for normal chow diet fed mice) in saline with the total volume of 100 μl was intraperitoneally (i.p) administrated and blood glucose levels were monitored at 15, 30, 60, 90 and 120 min. after the i.p. injection. For insulin tolerance test (ITT), mice were fasted for 6 hrs (8:00 a.m.–2:00 p.m.) and injected with recombinant human insulin from Eli Lilly (1 IU/kg bw for HFD fed mice and 1.5 IU/kg bw for lean mice). The blood glucose levels were measured before and 15, 30, 60, 90 and 120 min. after insulin administration.

### Statistical analysis

Data are presented as means ± S.E.M. Statistical significance was calculated by Student's *t*‐test or by two‐way anova. When anova indicated a significant difference among the groups, we compared the groups using a stricter criterion for statistical significance according to the Sidak's multiple comparison test. Significance was accepted at *P < 0.05, **P < 0.01 or ***P < 0.001.

### Ethnic statement for animal experiment

All animal experiments were approved by Institutional Animal Care and Use Committee (IACUC) at Boston Children's Hospital (BCH).

## Results

### Ablation of *Brd7* gene exhibits embryonic lethality

**Figure 1:**
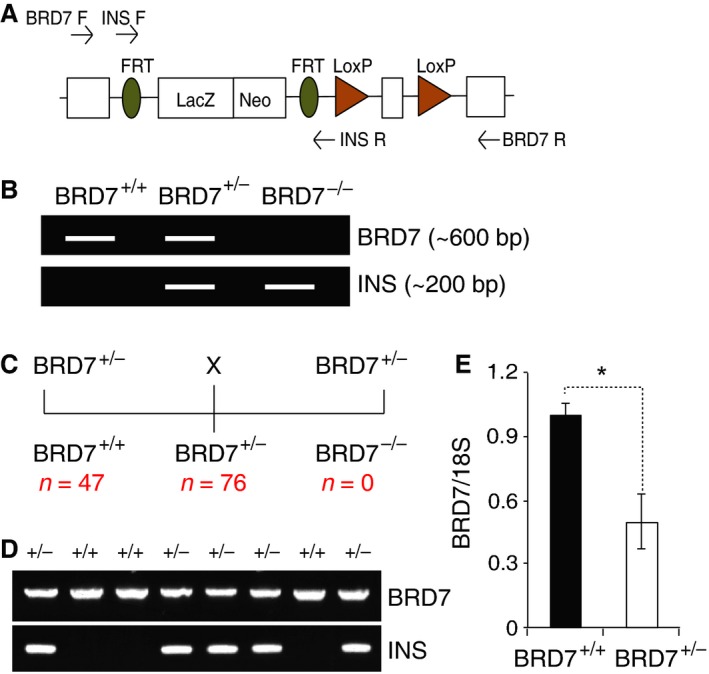
Genotype analysis of heterozygous BRD7 whole body knockout mice intercross. (**A**) Schematic diagram of *Brd7* gene targeting strategy. BRD7 primers are designed to flank the cassette and INS primers recognize FRT sites (INS: insert). (**B**) Schematic representation of PCR analysis of BRD7^+/+^, BRD7^+/−^, and BRD ^−/−^ mice. (**C**) Intercross progeny from BRD7^+/−^ mice. (**D**) Representative PCR analysis of offspring from BRD7^+/−^interbreeding. (**E**) *Brd7* mRNA levels of BRD7^+/+^ and BRD7^+/−^ mice. *18s* was used as a control gene. Significance was determined by Student's *t*‐test. *P < 0.05.

To investigate the function of BRD7 *in vivo*, we obtained heterozygous BRD7 whole body KO mouse line (BRD7^+/−^) from the European Conditional Mouse Mutagenesis Program. To generate homozygous BRD7 whole body KO (BRD7^−/−^), we intercrossed the BRD7^+/−^ mice. According to Mendelian inheritance, the expected genotypes of offspring are wild‐type (BRD7^+/+^), heterozygous BRD7 KO (BRD7^+/−^) and homozygous BRD7 KO (BRD7^−/−^) in 1:2:1 ratio, respectively. We identified the genotypes of offspring by performing PCR analysis of genomic DNA obtained from the tail. We used two sets of primer pairs to genotype the embryos: BRD7 primer pair, which recognizes wild‐type BRD7 allele, and INS (insert) primer pair, which is specific to the DNA sequence of the cassette that was inserted to generate BRD7 KO mouse line (Fig. 1A). As depicted in Figure 1B, BRD7^+/+^ mouse should result in amplification of a 607 base pair (bp) product when PCR is performed with the BRD7 primer pair and no band from PCR with the INS primer pair, since BRD7^+/+^ mice do not carry sequence complimentary to the latter primer pair. BRD7^+/−^ would test positive from both BRD7 and INS primer sets, and BRD7^−/−^ would amplify specific DNA only from the INS primer pair at a size of 204 bp. While BRD7^+/−^ mice were fertile with no obvious physical defect, genotyping of offspring from 20 breeding pairs identified 47 of BRD7^+/+^, 76 of BRD7^+/−^ and no BRD7^−/−^ (Fig. 1C). Figure 1D shows an example of genotyping results of one breeding pair, where it had 3 of BRD7^+/+^, 5 of BRD7^+/−^ and no BRD7^−/−^ offspring. This observation suggested that whole body deletion of BRD7 gene leads to embryonic lethality. BRD7^+/−^ mice displayed about 50% reduction in *Brd7* mRNA levels (Fig. 1E).

**Figure 2:**
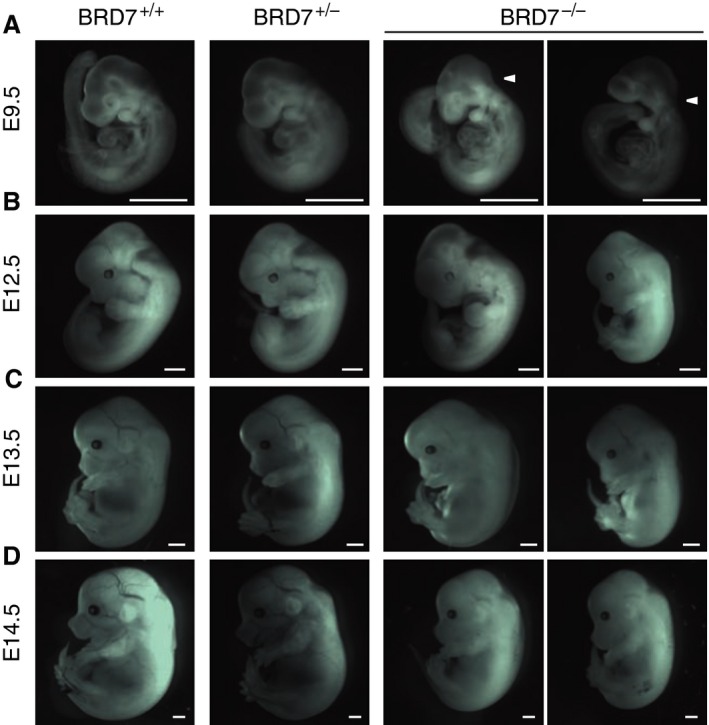
Morphological representations of embryos from heterozygous BRD7 whole body knockout mice intercross. Representative pictures of BRD7^+/+^, BRD7^+/−^, and BRD ^−/−^ embryos at (**A**) E9.5. (**B**) E12.5. (**C**) E13.5. (**D**) E14.5. Scale bars represent 1 mm.

**Figure 3:**
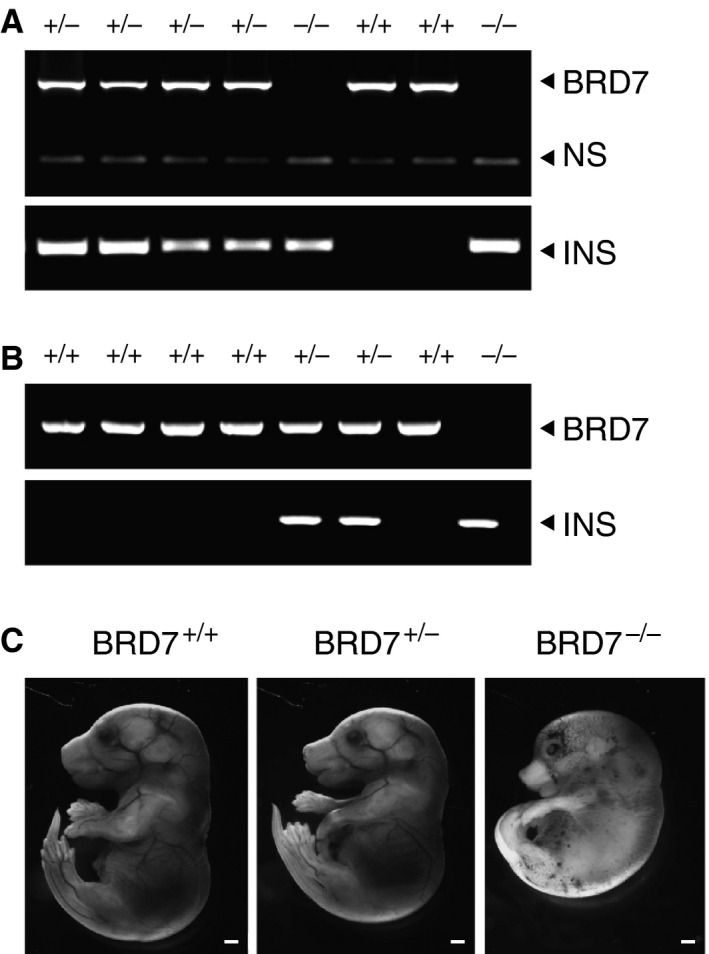
BRD7 embryos at mid‐ and late‐gestation. (**A**) Genotype analysis by PCR at E13.5 with BRD7 and INS primer sets. NS, non‐specific band. (**B**) Genotyping results of BRD7 embryos at E16.5. (**C**) Representative pictures of BRD7^+/+^, BRD7^+/−^ and BRD ^−/−^ embryos at E16.5. Scale bars represent 1 mm.

To characterize the timing of the embryonic lethality, we intercrossed BRD7^+/−^, and collected embryos at different stages of gestation. At embryonic day (E) 9.5, all embryos were vital with no major defects (Fig. 2A). We questioned about normal neural tube closure prior to this stage (arrowhead in Fig. 2A), as BRD7^−/−^ embryos displayed a slightly different appearance when compared to wild‐type mice or BRD7 heterozygous KO mice (Fig. 2A), however, we were still able to detect live BRD7^−/−^ embryos at E12.5 (Fig. 2B). Limbs are usually developed by E14. All three genotypes of embryos, BRD7^+/+^, BRD7^+/−^ and BRD7^−/−^, were *in utero* through E13.5, but BRD7^−/−^ embryos displayed delayed limb development (Fig. 2C). At E14.5, BRD7^−/−^ embryos were still detected, but exhibited growth retardation when compared to BRD7^+/+^ and BRD7^+/−^ embryos (Fig. 2D). Figure 3A depicts one example of genotyping from embryos at E13.5, which had identified 2 of BRD7^+/+^, 4 of BRD7^+/−^, and 2 of BRD7^−/−^ embryos. We then examined embryos at E16.5. The genotyping analysis showed 5 of BRD7^+/+^, 2 of BRD7^+/−^ and 1 of BRD7^−/−^ embryos (Fig. 3B). BRD7^−/−^ embryo at E16.5 was found dead *in utero* and displayed obvious abnormality with a smaller size and lack of proper limbs, blood vessels and organs development (Fig. 3C).

### Reduced BRD7 levels in the liver alone does not affect glucose metabolism

**Figure 4:**
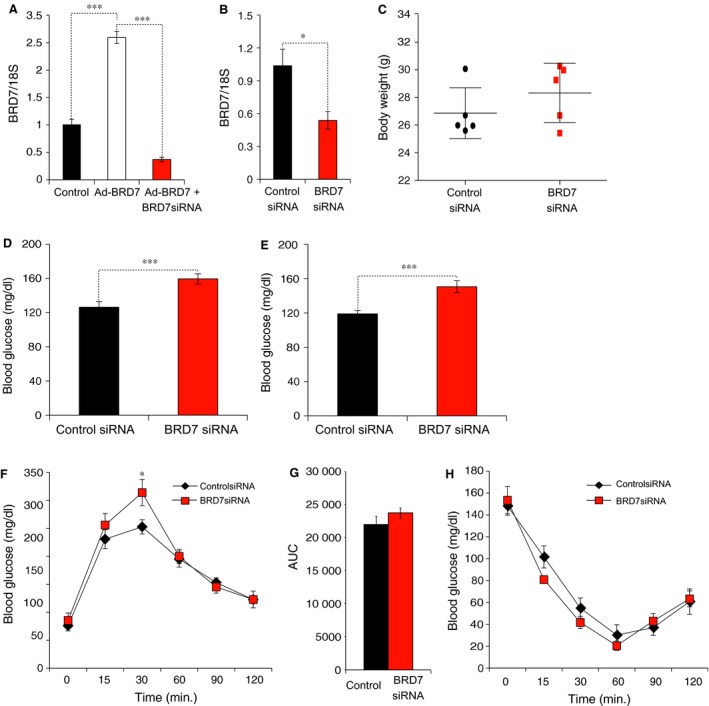
Effect of *Brd7* knockdown by siRNA. (**A**) mRNA levels of *Brd7* in MEFs that were infected with Ad‐BRD7 alone or together with BRD7siRNA. *18s* was used as a control gene. (**B**–**H**) Eight weeks old male wild‐type mice were injected with BRD7siRNA (*n* = 5) or scramble siRNA (*n* = 5) as a control through the tail vein. (**B**) mRNA levels of *Brd7* in the liver of siRNA injected mice on day 9 after injection. (**C**) Body weights on day 7 post‐injection. (**D**) Fed blood glucose levels (mg/dl) on day 3 after injection. (**E**) Fed blood glucose levels on day 5 of injection. (**F**) GTT on day 4 after injection and (**G**) area under the curve. (**H**) ITT on day 7 post‐injection. Significance was determined by two‐way anova and Sidak's multiple comparison in (**F** and **H**) or Student's *t*‐test in (**A**–**E** and **G**). *P < 0.05, ***P < 0.001.

We have previously reported that hepatic BRD7 expression levels are significantly reduced in obese and type 2 diabetic mice and acute overexpression of BRD7 in the liver through the tail vein injection of adenovirus that expresses BRD7 re‐establishes glucose homoeostasis 16. Therefore, we sought to investigate the knockdown effect of *Brd7*. For this purpose, we commercially obtained siRNA targeted against *Brd7*. We then infected MEFs with Ad‐BRD7 to induce its expression and added BRD7 siRNAs to silence the *Brd7* gene. Of five siRNA sequences, we then picked the one that the most efficiently silenced the *Brd7* gene (Fig. 4A). Consequently, we injected BRD7 siRNA and its control scramble siRNA into the tail vein of 8 weeks old wild‐type lean male mice. Administration of BRD7 siRNA yielded about a 50% reduction in *Brd7* mRNA levels in the liver on day 9 after injection (Fig. 4B) and it did not alter the bw of the animals when compared between the two groups (Fig. 4C). Three days after the administration of siRNAs, the blood glucose levels were significantly increased in BRD7‐specific siRNA‐group when compared to that of control group (Fig. 4D). The elevated blood glucose levels in BRD7 siRNA group persisted for 5 days (Fig. 4E). Administration of BRD7 siRNA leads to mild disturbance in glucose tolerance (Fig. 4F), but AUC did not show a significant difference between the two groups (Fig. 4G). Insulin tolerance test did not display any significant difference between the two groups (Fig. 4H).

**Figure 5:**
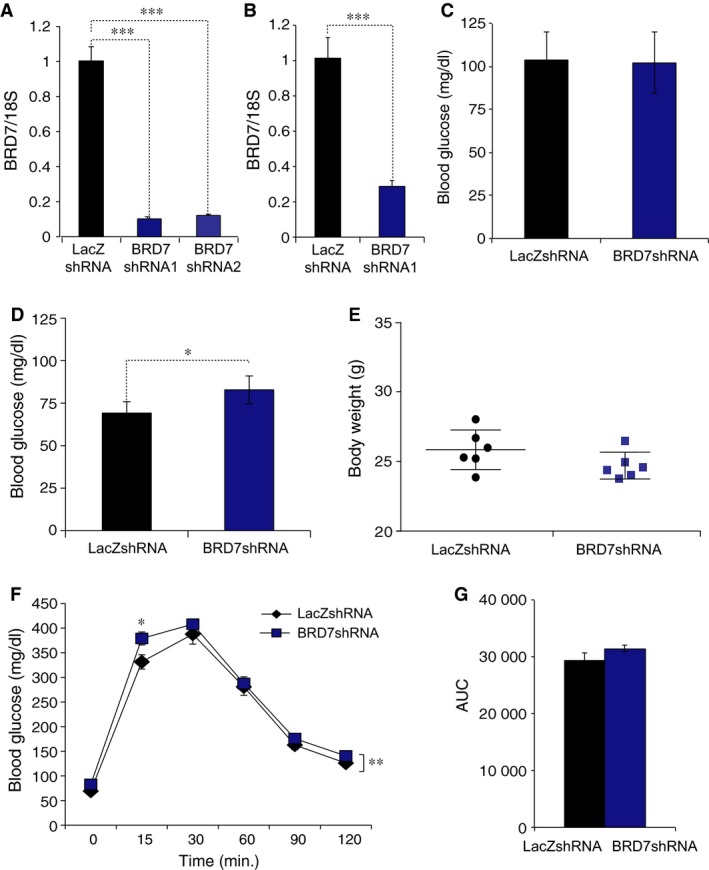
Effect of adenovirus‐mediated *Brd7* knockdown. (**A**) mRNA levels of *Brd7* in MEFs that were infected with Ad‐BRD7shRNA (*n* = 5) or Ad‐LacZshRNA (*n* = 5) as a control. *18s* was used as a control to normalize. (**B**–**H**) Eight weeks old male wild‐type mice were injected with Ad‐BRD7shRNA or Ad‐LacZshRNA as a control through the tail vein at the 1 × 10^9^ plaque forming units (PFU) per g bw. (**B**) *Brd7* mRNA levels in the liver of Ad‐LacZshRNA‐ and Ad‐BRD7shRNA‐injected groups on day 8 after injection. (**C**) Fed blood glucose levels on day 3 post‐injection. (**D**) Blood glucose concentrations on day 4 after 16 hrs of fasting. (**E**) Body weights on day 5 after injection. **(F)** GTT on day 4 and (**G**) AUC. Significance was determined by two‐way anova and Sidak's multiple comparisons in (**F**) or Student's *t*‐test in (**A**–**E** and **G**). *P < 0.05, **P < 0.01, ***P < 0.001.

**Figure 6:**
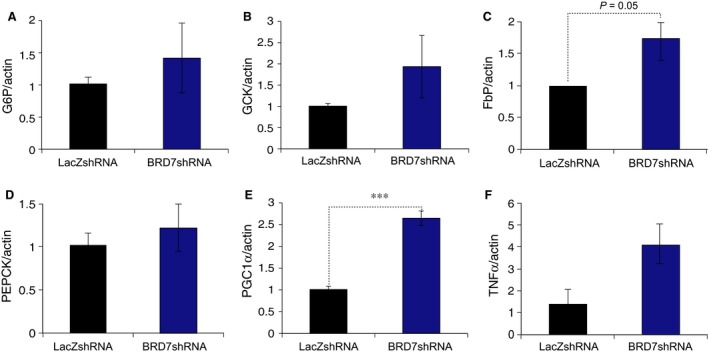
Effect of *Brd7* downregulation on gene expressions. Eight weeks old male wild‐type mice were subjected to injection of Ad‐BRD7shRNA (*n* = 5) or Ad‐LacZshRNA (*n* = 5) through the tail vein at the 1 × 10^9^ plaque forming units (PFU) per g bw. RNA was isolated from the liver tissue and qPCR was performed. Relative mRNA levels of (**A**) *G6P*. (**B**) *GCK*. (**C**) *FbP*. (**D**) *PEPCK*. (**E**) *PGC1*α. (**F**) *TNF*‐α. β*‐actin* was used as an internal control to normalize. Significance was determined by Student's *t*‐test. ***P < 0.001.

To further down‐regulate *Brd7* gene, we constructed adenovirus that carries BRD7‐specific shRNA (Ad‐BRD7shRNA) and used LacZshRNA carrying adenovirus (Ad‐LacZshRNA) as a control. We infected MEFs with two different Ad‐BRD7shRNAs, and confirmed the efficiency of Ad‐BRD7shRNA (Fig. 5A). We then injected 8 weeks old wild‐type male mice with 1 × 10^9^ plaque forming units per g bw of Ad‐BRD7shRNA or Ad‐LacZshRNA. qPCR analysis from mRNA obtained from the liver of adenovirus injected group showed approximately 70% reduction in *Brd7* mRNA levels on day 8 after injection (Fig. 5B). There was no difference in blood glucose concentrations on day 3 after injection (Fig. 5C), but it started to show the knockdown effect from day 4 post‐injection; there was a slight but significant increase in the blood glucose level in the Ad‐BRD7shRNA‐injected group when compared to the control group (Fig. 5D). There was no significant difference in body weights in the two groups (Fig. 5E). Glucose tolerance test performed on day 4 after injection showed a trend of developing glucose intolerance (Fig. 5F), but AUC did not display a significant difference between Ad‐BRD7shRNA‐ and Ad‐LacZshRNA‐injected groups (Fig. 5G). In line with disturbed glucose metabolism, gene expression levels of *G6P*,* GCK*,* FbP*,* PEPCK* and *PGC1*α were increased in Ad‐BRD7shRNA‐injected group when compared to those in Ad‐LacZshRNA‐injected group (Fig. 6A–E). Lastly, the knockdown of *Brd7* led to increase in *TNF*‐α, however, it did not achieve significance (Fig. 6F).

### Reduction in BRD7 levels and HFD challenge result in disturbed glucose homoeostasis

**Figure 7:**
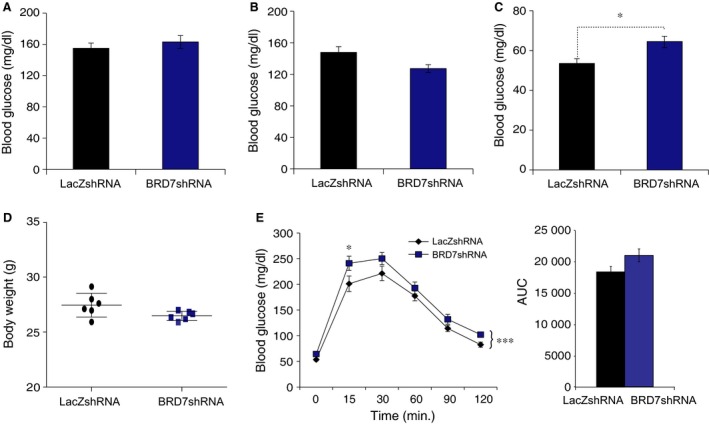
Disturbed glucose tolerance after knockdown of *Brd7* and a high fat diet (HFD) feeding. Wild‐type male mice were fed on HFD for 3 weeks, followed by tail vein injection of Ad‐BRD7shRNA (*n* = 6) or Ad‐LacZshRNA (*n* = 6). (**A**) Fed blood glucose concentrations on day 3 after injection. (**B**) Fed blood glucose levels on day 6 after injection. (**C**) Blood glucose levels on day 4 of injection at 16 hours of fasting. (**D**) Body weights on day 7. (**E**) GTT on day 4 (left) and AUC (right). Significance in was determined by two‐way anova and Sidak's multiple comparison in (**E**) or Student's *t*‐test in (**A**–**E**). *P < 0.05, ***P < 0.001.

Obesity is accompanied by hyperinsulinemia and hyperglycaemia. We have previously reported HFD‐induced obesity leads to reduction in hepatic BRD7 expression levels 16. Therefore, we sought to investigate whether adenovirus‐mediated reduction in hepatic BRD7 levels in combination with HFD challenge would have a synergetic effect and result in disturbed glucose homoeostasis. For this purpose, we first placed 3.5 weeks old male wild‐type mice on a HFD for 3 weeks, and injected them with Ad‐BRD7shRNA or Ad‐LacZshRNA through the tail vein. There was no difference in fed blood glucose level on day 3 and 6 after injection between the two groups (Fig. 7A and B). However, the Ad‐BRD7shRNA‐injected group had higher blood glucose levels than those in Ad‐LacZshRNA‐injected group at 16 hrs fasted state on day 4 after the injection (Fig. 7C). There was no difference in the bw between the two groups (Fig. 7D). Glucose tolerance test was performed on day 4 post‐injection, and the rate in the Ad‐BRD7shRNA‐injected group revealed a significant disturbance in the glucose disposal rate (Fig. 7E).

**Figure 8:**
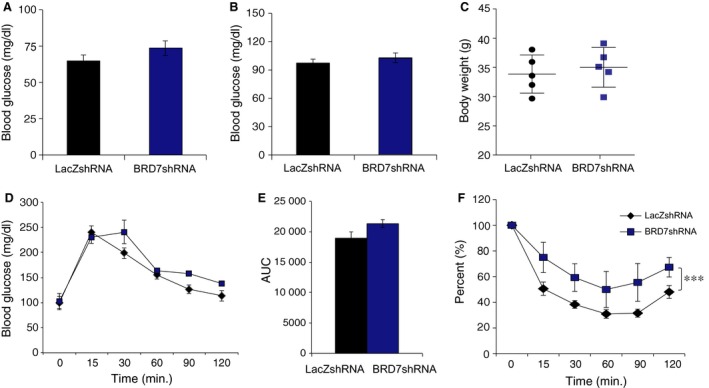
Decreased insulin sensitivity after *Brd7* knockdown in HFD‐induced obese mice. Wild‐type male mice were fed on HFD for 11 week, then subjected to tail vein injection of Ad‐BRD7shRNA (*n* = 5) or Ad‐LacZshRNA (*n* = 5). (**A**) Blood glucose levels on day 5 of injection at 16 hours of fasted state. (**B**) Fed blood glucose levels on day 6 of injection. (**C**) Body weights on day 9. (**D**) GTT on day 9 and (**E**) AUC. (**F**) ITT on day 7. Significance was determined by two‐way anova and Sidak's multiple comparison in (**D** and **F**) or Student's *t*‐test in (**A**–**C** and **E**). ***P < 0.001.

We then extended the period of HFD challenge to 11 weeks before injecting the mice with Ad‐BRD7shRNA or Ad‐LacZshRNA through the tail vein. On day 5 after the injection, 16 hrs fasted blood glucose levels also did not show any difference between Ad‐BRD7shRNA‐ and Ad‐LacZshRNA‐injected groups (Fig. 8A). Fed glucose levels on day 6 post‐injection did not display any difference between the two groups (Fig. 8B). There was no difference in bw between the groups (Fig. 8C). Glucose tolerance test was performed at day 9 after the injection, and the result showed no major disturbance in the glucose disposal rate in the Ad‐BRD7shRNA‐injected group when compared to Ad‐LacZshRNA‐injected group (Fig. 8D and E). However, ITT at day 7 revealed that whole body insulin sensitivity was decreased in Ad‐BRD7shRNA‐injected group (Fig. 8F).

## Discussion

The role of BRD7 has been implicated in cancers however, not much is known about its function in glucose metabolism. We have recently shown that acute up‐regulation of BRD7 improves glucose tolerance in obese mouse models 16. This led us to further investigate the role of BRD7 in the maintenance of metabolic homoeostasis. First, we initially intended to generate homozygous BRD7 whole body KO mice to understand the biological role of BRD7 in *in vivo* setting. To do this, we obtained heterozygous BRD7 KO mouse model and intercrossed them. While more detailed investigation is required to understand the cause, we found that homozygous BRD7 KO mice are embryonic lethal prior to embryonic day16.5.

Reduction in hepatic BRD7 expression levels has been observed in obese and type 2 diabetic mice in our previous study 16, implicating its potential role in the development of glucose intolerance and insulin resistance in obesity. To investigate the effect of *Brd7* reduction in glucose metabolism, we used siRNA and shRNA systems to knockdown the *Brd7* gene in wild‐type lean mice. Partial deletion of *Brd7* in the liver has led to mild decreased glucose tolerance and insulin sensitivity in the lean mice. It is possible that remaining BRD7 protein was working to maintain glucose homoeostasis. In fact, there are many examples of genes with a critical function, where total KO leads to embryonic lethality, yet heterozygous KO mice do not display any obvious phenotype. For instance, XBP1 deletion leads to embryonic lethality 19. However, a null mutation of XBP1 in one allele does not display any particular phenotype, especially in terms of bw and glucose metabolism, until they are challenged with a HFD feeding 20. They display glucose intolerance and insulin resistance when they were fed on a HFD 20. Another example can be seen in BRCA1, a *tumor* suppressor gene, which was recently reported to interact with BRD7 15. Mutation in BRCA1 accounts for half of all familial breast cancer cases 21. It was shown that homozygous BRCA1 KO mice are embryonic lethal 22, however, heterozygous BRCA1 KO mice do not display any phenotypic defect and they do not develop *tumors* 23. Meanwhile, BRCA1 and p53 double deficient mice developed breast cancers that are phenotypically similar to *tumors* from human BRCA1 mutations 24.

Therefore, we sought to investigate whether BRD7 deficiency in combination with obesity might have a synergistic effect in disturbance of glucose homeostasis. For this purpose, we have challenged wild‐type mice on a HFD feeding before introducing Ad‐BRD7shRNA. *Brd7* knockdown in combination with a HFD led to further disturbed glucose homoeostasis and insulin resistance. Meanwhile, GTT result from Ad‐BRD7shRNA‐injected group that was placed on a HFD for 11 weeks did not show significant difference compared to that from Ad‐LacZshRNA‐injected group. It is probably because HFD feeding itself led to a reduction in BRD7 expression in both groups, and therefore, it is likely that even Ad‐LacZshRNA‐injected group had reduced *Brd7* expression in the liver after 11 weeks of HFD, which is also accompanied by disturbance in glucose metabolism.

It was shown that BRD7 binds to p85s, the regulatory subunits of PI3K through its C‐terminus region 17. The sequence of this binding site is highly conserved from *Homo sapiens* to *Caenorhabditis elegans* 17. This degree of conservation throughout a wide range of species implies that BRD7 plays an essential role in the insulin signalling pathway and glucose homeostasis. In conclusion, our study suggests that BRD7 plays a critical role in embryogenesis and further helps understand the phenotype of BRD7 deficiency in glucose metabolism.
