# Corrigendum

**DOI:** 10.1111/jcmm.13187

**Published:** 2017-05-15

In Yoo Kim et al. 1, page 1563, an error occurred under the section of Quantitative real‐time PCR, as the qPCR primer sequence for BRD7 is listed incorrectly. The correct primer sequence should be as stated below:

BRD7 Forward 5′‐GAGGCTGAGGTGTTCCAGAG‐3′
BRD7 Reverse 5′‐TCACCTGGAGTCACTTGCTG‐3′

The authors confirm that there are no conflict of interests and wishes to apologize for any misunderstanding or inconvenience caused.
